# An Unusual Location of Neuroendocrine Tumour: Primary Hepatic Origin

**DOI:** 10.1155/2015/461420

**Published:** 2015-05-07

**Authors:** A. Bahar Ceyran, A. Tarık Artış, Serkan Şenol, Bengü Çobanoğlu Şimşek

**Affiliations:** ^1^Department of Pathology, Medeniyet University Medical School Göztepe Research and Training Hospital, Kadıkoy, 34730 Istanbul, Turkey; ^2^Department of General Surgery, Medeniyet University Medical School Göztepe Research and Training Hospital, Kadıkoy, 34730 Istanbul, Turkey

## Abstract

Although neuroendocrine tumours (NETs) of primary hepatic origin are extremely rare, most of NETs present with liver metastasis. When a NET is found in the liver, it must be treated to exclude metastasis from extrahepatic primary sites. The patient was a 38-year-old female. Abdominal ultrasound showed an 8 cm tumour in liver during a routine examination. Liver biopsy was done. The tumour was first considered a metastatic hepatic tumour on histopathological examination. No clues to the origin of a primary tumour were found. Upper and lower endoscopy of the GI tract and chest CT were performed to search for a primary tumour and were negative for any tumour. One month later, more extensive areas of the tumour were seen on histopathological examination of second liver biopsy with the same morphologic characteristics as the first biopsy. Immunohistochemically, there was positive staining for synaptophysin, CD 56, and S-100 in the tumour cells. These findings suggested the diagnosis of NET. The diagnosis of primary liver NET was considered in a multidisciplinary meeting. Then, left hepatectomy was performed. The final pathologic diagnosis of the tumour in the resected liver specimen was Grade II NET. The patient was doing well at postoperative 28-month follow-up.

## 1. Introduction

Neuroendocrine tumours (NETs) are rare tumours that originate from dispersed neuroendocrine cells distributed throughout the body. NETs can be either functioning or nonfunctioning. Functioning NETs produce vasoactive hormones such as serotonin, gastrin, insulin, glucagon, and somatostatin, which may cause carcinoid syndrome [1].

NETs are also called carcinoid tumours. According to the World Health Organisation (WHO) 2000 classification, classic carcinoids were classified as well-differentiated endocrine tumours; however, in the WHO 2010 classification, they were classified as NET Grade I. Well-differentiated endocrine carcinomas were classified as NET Grade II, and poorly differentiated endocrine carcinomas were classified as neuroendocrine carcinomas (NECs), large-cell or small-cell type [2].

NETs are seen most frequently in the bronchopulmonary tree or gastrointestinal tract, but they may be seen in nearly every organ [3, 4]. Primary NETs are most commonly seen in the lung, stomach, ileum, duodenum, jejunum, pancreas, Meckel, appendix, rectum, colon, breast, and ovaries [5]. Gastroenteropancreatic (GEP) NETs are the largest group, accounting for 2% of all gastrointestinal tumours [3, 5].

The incidence of NETs in the United States is 2-3/100.000 per year [3, 5, 6]. However, the majority of NETs are clinically silent, so the real incidence might be higher [3]. Primary hepatic NETs are extremely rare [1, 3]. According to our literature search, fewer than 100 primary hepatic NET cases were reported, mostly as case reports [7]. The largest reported groups included 11, 9, and 8 patients, respectively [1, 7]. In addition, most NET cases present with liver metastasis. Therefore, when an NET is found in the liver, it must be treated with great care to exclude metastasis from extrahepatic primary sites, as there is much more common occurrence [1, 3, 5, 7]. In the literature, the first primary hepatic NET was reported by Edmonson in 1958 [3]. Herein, we report a new case of primary hepatic NET.

## 2. Case Report

**Figure 1:**
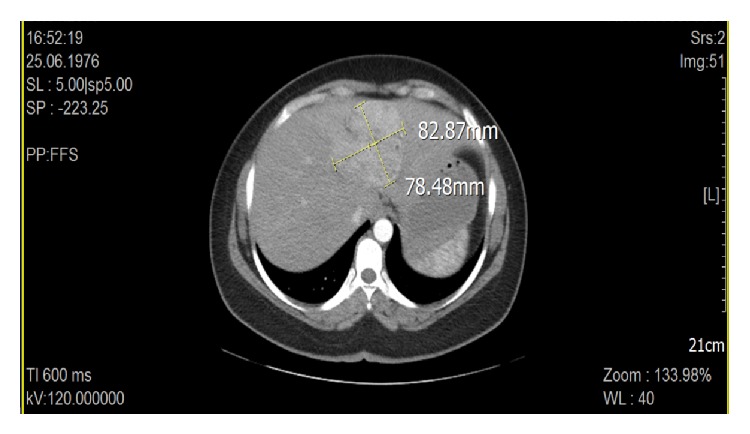
CT appearance of the tumor in the liver (CT image of hepatic mass).

A 38-year-old female complained of abdominal discomfort and pain. Her past medical history was not significant. Physical examination and all biochemical laboratory results were within normal limits, including liver function tests, tumour markers (CA19-9, CEA, AFP, and CA15-3), and chromogranin A. Abdominal ultrasound showed an 8 cm tumour in liver segment IV. Characteristics of abdominal CT (Figure 1) and MRI led to a diagnosis of either fibrolamellar hepatocellular carcinoma (HCC) or focal nodular hyperplasia (FNH).

**Figure 2:**
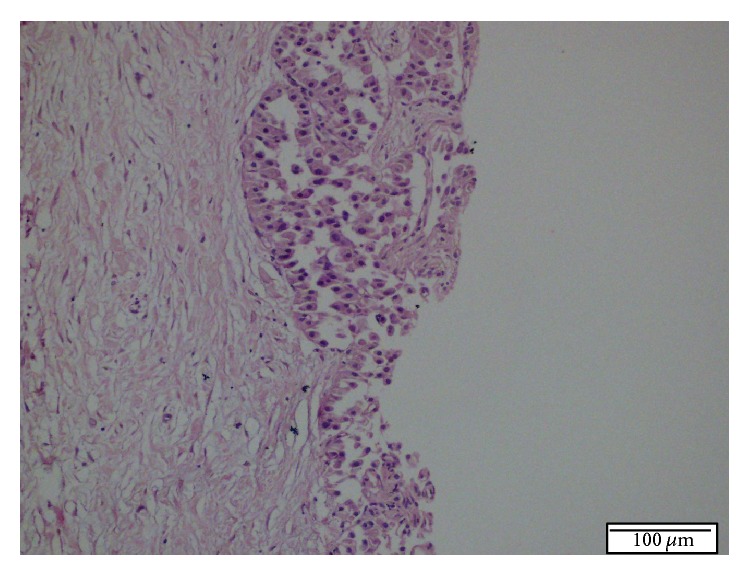
Histopathologic appearance of tumoral lesion in first Tru-Cut biopsy. Microscopically, a tumor consists of epithelial cells with round, basophilic, uniform nucleus, inconspicuous nucleoli, and pale eosinophilic granulated cytoplasm. The architectural pattern was trabecular, ribbonlike, and partly acinar. No significant cytologic atypia, mitosis, and necrosis were present. H.E. ×200.

Liver biopsy was done. Histopathological examination revealed a tumour that consisted of epithelial cells with round basophilic uniform nuclei, inconspicuous nucleoli, and pale eosinophilic granulated cytoplasm. The architectural pattern was trabecular, ribbonlike, and partly acinar. No significant cytologic atypia, mitosis, or necrosis was present (Figure 2). Immunohistochemically, there was negative staining for Heppar-1, CK 19, and CK 20.

Morphology was not characteristic for HCC, and at first the tumour was considered a metastatic hepatic tumour. No clues to the origin of a primary tumour were found. Further evaluations and examinations were undertaken to search for a primary tumour. Chest CT and upper and lower endoscopy of the GI tract were performed and were negative for any tumour.

One month later, a second liver biopsy was done, and histopathological examination showed the same morphologic characteristics as the first biopsy, but more extensive areas of the tumour were seen. Immunohistochemically, there was diffuse and strongly positive staining for synaptophysin, CD 56, and S-100 in the tumour cells. These findings supported the diagnosis of NET. Additionally, there was negative staining for Heppar-1, AFP, pCEA, and TTF-1 and positive staining for CK 8/18, and the Ki 67 proliferation index was 5-6%.

Given these findings, the pathological diagnosis was Grade II NET. At first, it was considered a metastatic NET, so Gallium 68 peptide PET scintigraphy was done to search for a primary tumour. But this test was negative, and another tumour could not be detected. A multidisciplinary meeting confirmed the diagnosis of primary liver NET, and laparotomy was decided upon for surgical resection.

The patient underwent laparotomy, and, during the exploration, intraoperative ultrasound was done, finding no other tumour. An anatomical left hepatectomy was performed. The patient's postoperative course was uneventful, and she was discharged on postoperative day 10.

**Figure 3:**
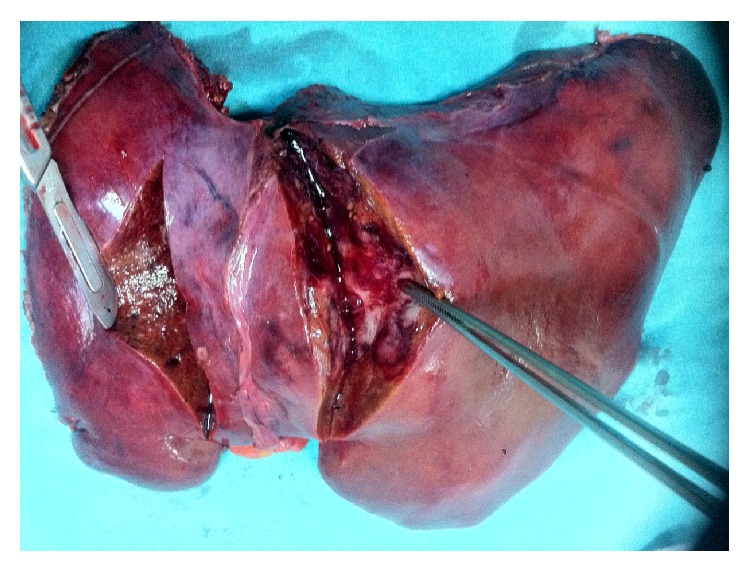
Gross appearance of the resected liver specimen.

An 8 cm tumour was found upon macroscopic evaluation of the specimen (Figure 3). The tumour was strongly and diffusely positive for chromogranin A. Three mitoses were present per 10 high power fields.

**Figure 4:**
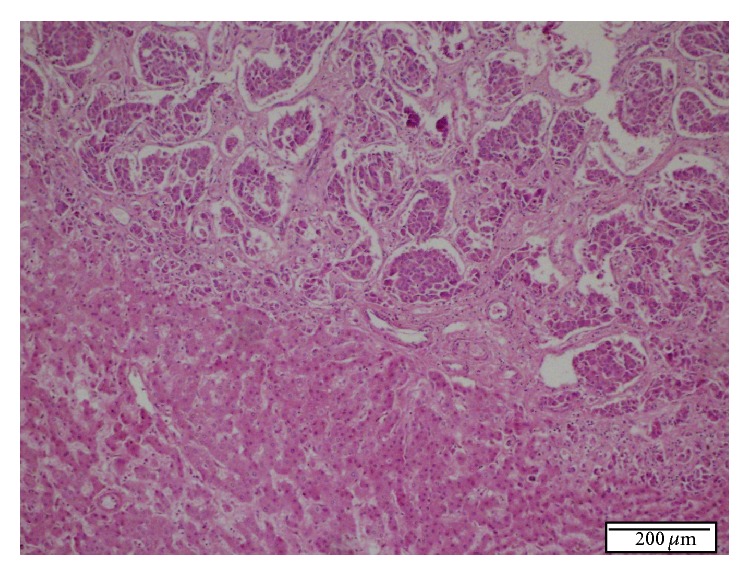
Histopathologic appearance of tumoral lesion in the resected liver specimen. Microscopic characteristics are similar in first and second Tru-Cut biopsies. H.E. ×100.

**Figure 5:**
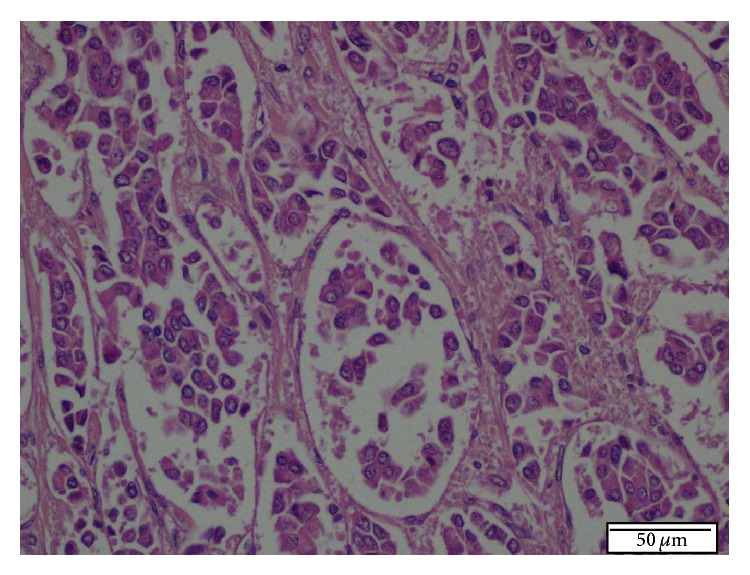
Microscopic appearance of tumoral lesion in the resected liver specimen at higher magnification. H.E. ×400.

**Figure 6:**
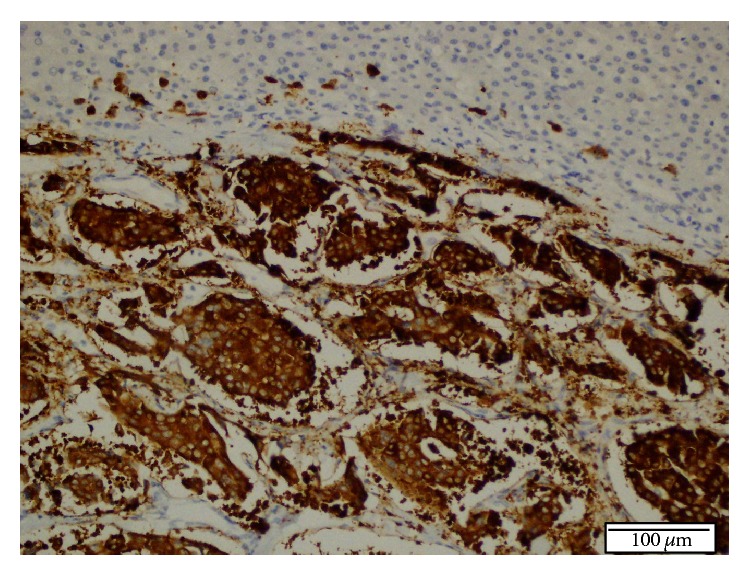
Diffuse, strong immunoreactivity for synaptophysin in tumor cells in the resected liver specimen. Immunohistochemical (IHC) staining, ×200.

**Figure 7:**
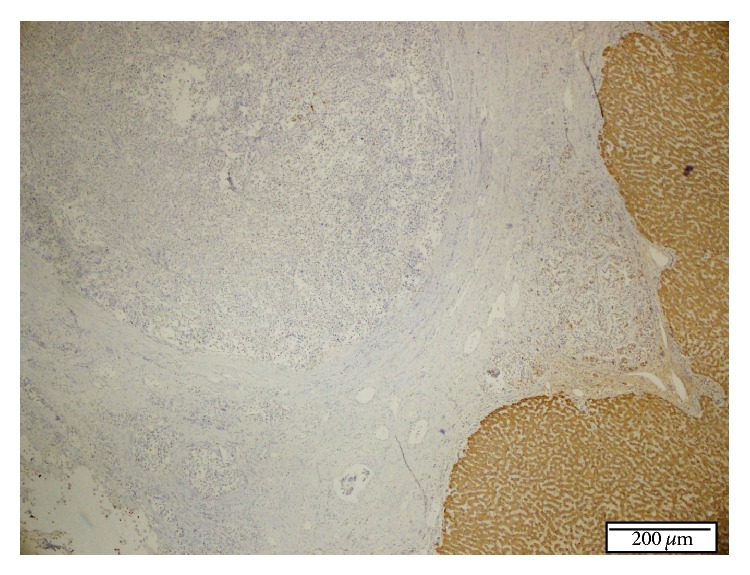
Negative immunoreactivity for Heppar-1 in tumor cells; in contrast positive immunoreactivity occurs in normal liver cells in the resected liver specimen. IHC staining, ×40.

**Figure 8:**
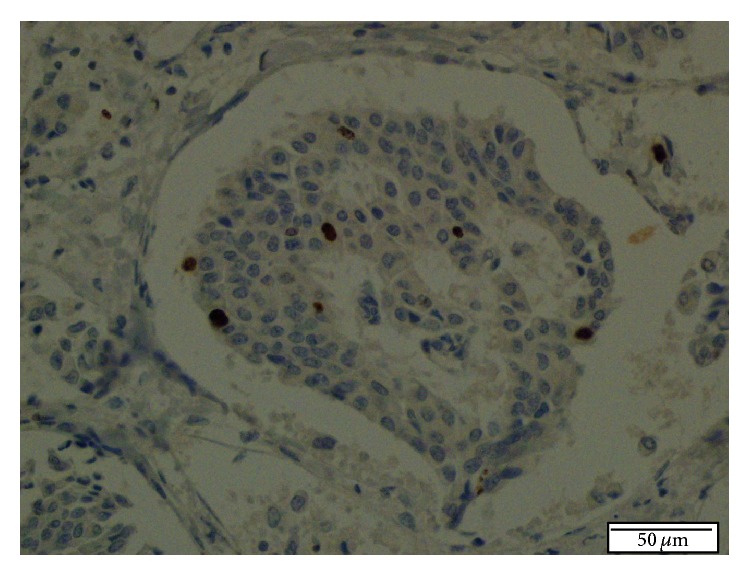
Ki 67 proliferation index is 5-6% in tumor cells. IHC staining, ×400.

The final pathologic diagnosis was Grade II NET (Figures 4–8). Surgical margins were free of tumour. Adjuvant therapy was not indicated by the oncological consultation. Recently, the patient was doing well at her 28-month postoperative follow-up.

## 3. Discussion

Primary hepatic NETs characteristically grow slowly and become clinically evident only at an advanced stage [8]. In most cases, they are incidentally detected, as they are nonfunctional [3]. Mean age is 49.8, with a slight female predominance [3, 7]. In a group of 53 primary hepatic NETs, the male/female ratio was 20/33 [9]. In general, such tumours are located in the central liver. Our patient was 38-year-old female and had nonspecific symptoms regarding the liver tumour, which was centrally located in the liver and found incidentally, as reported in the literature.

The histogenesis of primary hepatic NET is not clear. According to one theory, it originates from neuroendocrine cells scattered in the intrahepatic biliary epithelium. A second theory speculates that chronic inflammation in the biliary system causes intestinal metaplasia and NET [3]. In our case, there is no chronic liver or biliary disease or history of chronic inflammation. Therefore, this case does not correlate with the second theory.

Symptoms of primary hepatic NET differ from those of HCC and other hepatic tumours. Clinical manifestation may vary according to carcinoid syndrome presence. Early and definitive diagnosis prior to biopsy or resection is difficult due to nonspecific clinical presentation and rarity [1]. There is an interesting case of a 9-year-old patient who has Cushing's syndrome with a hepatic tumour identified by ultrasound as haemangioma and reported with an ectopic ACTH producing a primary hepatic NET. In addition, in the literature, there were fewer than 30 primary hepatic gastronomies, some of which were reported as Zollinger-Ellison syndrome [10].

Misdiagnosis is frequent prior to pathological examination for primary hepatic NETs. CT scan and MRI are two useful tools for diagnosis and treatment planning [1]. The characteristics on dynamic MRI are dominant hypervascular large mass with satellite nodules. However, definitive diagnosis is not possible with these findings alone. Furthermore, for both primary and metastatic NETs, radiologic features are similar [3, 4]. In our case, MRI findings were consistent with both HCC and FNH. Definitive diagnosis of NET was possible only after hepatic biopsy. Therefore, biopsy and pathological examination are the gold standard for diagnosis [1].

On histopathological examination, NETs (classic carcinoid tumours) are composed of uniform round or polygonal cells having monotonous round centrally located nuclei with finely stippled chromatin, small nucleoli and wide pale eosinophilic granulated cytoplasm, infrequent mitosis, and no necrosis. The tumour may have various patterns such as solid, trabecular, ribbonlike, or acinar. Poorly differentiated malignant forms of these tumours are either (1) large-cell NECs (atypical carcinoids), which consist of large atypical cells with marked cellular pleomorphism, large irregular hyperchromatic nuclei, prominent nucleoli, and tumour necrosis and mitosis or (2) small-cell NECs, which consist of small atypical cells with extremely high nucleocytoplasmic ratios, homogeneously dark nuclear chromatin, inconspicuous nucleoli, scanty cytoplasm, and numerous mitoses [9].

The WHO classification used for the histopathological differentiation of NET and NEC was revised in 2010. According to the new classification, mitosis number and Ki 67 proliferation index are important determining factors for grading and classifying NETs. In histopathological examination of primary hepatic NETs, the concomitance of HCC and NET is extremely rare but can be either the combined type or the collusion type. Our case was diagnosed as Grade II NET according to the 2010 WHO NET classification, and pure NET areas were observed.

Long-term follow-up for treatment of primary NETs has not been reported on sufficiently. However, the prognosis seems favourable. Aggressive treatment is not required [7]. When lymph node and distant metastases are absent, liver resection is adequate for treatment. If lymph node metastasis is present, adjuvant chemotherapy may be needed [8]. Transarterial chemoembolization or radiotherapy treatments also have been reported as effective treatment modalities [8]. Five-year survival and recurrence rates after surgical resection have been reported as 74% and 18%, respectively [9]. For treating our case, left hepatectomy was performed, since there were no distant or lymph node metastases. The patient is on a regular follow-up schedule and at 28 months after surgery is healthy, without recurrence.

## 4. Conclusions

When a hepatic tumour is considered to be NET, first a detailed systemic search for a primary tumour is mandatory to exclude metastatic NET, as primary hepatic NETs are very rare compared to metastatic ones. Upon exact diagnosis of primary hepatic NET, liver resection is the treatment of choice for successful long-term treatment. However, further studies with larger numbers of patients and long-term follow-up are needed to better understand the outcomes and nature of this rare type of tumour.
